# Ewing sarcoma-related pain: potential role of medical cannabis monotherapy in symptom management – a case report

**DOI:** 10.1186/s42238-026-00388-x

**Published:** 2026-01-23

**Authors:** Cesare De Virgilio Suglia, Felice Antonio Spaccavento, Fabio Turco, Angela De Trizio, Rossella Giannuzzi, Silvio Tafuri

**Affiliations:** 1 Department of Precision and Regenerative Medicine and Ionian Area (DiMePreJ), University of Bari Aldo Moro, Bari, Italy; 2 Palliative Care Unit, ASL Bari (Bari Local Health Trust), Bari, Italy; 3 Cannabiscientia SA, Lugano, Switzerland; 4 Interdisciplinary Department of Medicine (DIM), University of Bari Aldo Moro, Bari, Italy

**Keywords:** Ewing sarcoma, Medical cannabis, Pain management, Opioid sparing, Refractory pain, Cancer pain, Periprosthetic osteomyelitis, Cannabinoids, THC

## Abstract

**Background:**

Persistent, multimodal cancer pain remains a challenge, particularly in long-term survivors facing treatment-related complications. The management of high-dose opioid dependence concurrent with chronic, multi-drug resistant (MDR) periprosthetic infection presents a critical unmet need. This case reports the potential use and sustained efficacy of medical cannabis monotherapy, highlighting an unexpected temporal association with the resolution of inflammatory and infectious symptoms in a highly complex oncologic setting.

**Case presentation:**

A 27-year-old male, a long-term survivor of high-risk Ewing Sarcoma of the proximal tibia, presented with intractable mixed pain (VAS 9–10) secondary to chronic, recurrent MDR periprosthetic osteomyelitis and multiple surgical revisions (2013–2024). Despite continuous use of high-dose opioids (up to 120 mg/day morphine equivalents), pain levels remained moderate-to-severe (VAS 6–7) and functional status was poor. The patient had previously found temporary relief with self-administered cannabis. In January 2025, after refusing limb amputation, supervised medical cannabis therapy (Bedrocan^®^, 22% THC, 1% CBD, 1 g/day) was initiated. Pain levels gradually stabilized at VAS 2–3, coinciding with complete opioid discontinuation within four weeks. Over nine months of follow-up, the patient maintained full autonomy and an active lifestyle. Notably, sustained cannabis monotherapy was associated with the complete closure of the chronic draining fistula and a reduction in systemic inflammatory markers (CRP from 9.6 to 2.3 mg/dL). No significant adverse effects were reported.

**Conclusions:**

This case suggests that THC-rich medical cannabis may represent a feasible strategy for achieving opioid-free analgesia in selected patients with refractory oncologic pain. While causality cannot be established from a single observation, the correlation between cannabis initiation and the resolution of severe chronic inflammatory and infectious symptoms is intriguing and suggests a potential pleiotropic role extending beyond traditional pain management. While these findings align with emerging evidence highlighting the potent immunomodulatory and anti-inflammatory properties of cannabinoids, they contrast with some recent neutral meta-analyses in broader populations, an this would justify warrant urgent controlled investigation into the potential mechanisms of cannabinoids in complex inflammatory pain states and their role as a possible adjunct in managing long-term oncological complications.

## Introduction

Resistant cancer pain remains a pervasive and multifactorial problem, affecting a large part of oncological patients, with an estimated pooled prevalence of 45% across all disease stages and reaching more than 54% among patients receiving palliative treatment (Lawson et al. 2025). Pain is common for oncological patients and its impact on quality of life could be devastating. Mechanisms underlying cancer pain are complex and include direct tumor invasion of bone, viscera, or neural structures; tissue ischemia and inflammation sustained by the tumor microenvironment; and treatment-related toxicities such as chemotherapy-induced peripheral neuropathy, radiation fibrosis, or post-surgical nerve injury (van den Beuken- Everdingen et al. 2016; Schmidt 2014).

Current strategies are based on the use of opioids, non-steroidal anti-inflammatory drugs, and adjuvant agents such as antidepressants or anticonvulsants. In particular, the amount and quality of evidence around the use of opioids for treating cancer pain is disappointingly low as they often provide incomplete relief or lead to adverse effects that compromise long-term adherence (Caraceni et al. 2012; Wiffen et al. 2017; Fallon et al. 2018). Studies estimate that around one-third of patients continue to experience moderate-to-severe pain despite optimized therapy. Because these symptoms lead to decreased quality of life and affect compliance with therapies, there is a pressing need for alternative approaches that address both nociceptive and neuropathic components without increasing toxicity (van den Beuken- Everdingen et al. 2016; Pachman et al. 2012).

In recent years, medical cannabis has attracted increasing attention as an adjunctive option for pain management in oncology. A 2024 Guideline from the American Society of Clinical Oncology (ASCO) provided strategies for open, nonjudgmental communication between clinicians and adults with cancer about the use of cannabis and/or cannabinoids, while advising against its use as a cancer-directed therapy outside clinical trials. The panel judged the best-supported indication to be “refractory chemotherapy-induced nausea and vomiting” added to standard antiemetic regimens, with uncertainty persisting for other supportive-care outcomes including pain (Braun et al. 2024).

A 2025 metanalysis encompassing 39,767 data points related to cannabis and various health outcomes indicates a strong and growing consensus within the scientific community regarding the therapeutic benefits of cannabis, particularly in the context of cancer (Castle et al. 2025).

However, the clinical evidence remains conflicting. A 2012 trial on 263 patients demonstrated that nabiximols were more effective than placebo at low-to-medium doses (Portenoy et al. 2012). Conversely, a 2018 phase III study on 397 patients showed no difference between nabiximols and placebo in median pain score improvements, although benefits were noted in secondary quality-of-life outcomes (Lichtman et al. 2018). Furthermore, a 2023 Cochrane review indicated moderate-certainty evidence that THC and nabiximols may not relieve opioid-refractory cancer pain in broad populations, with single-dose synthetic THC analogues or CBD providing no additional benefit (Häuser et al. 2023). These discrepancies suggest that the efficacy of cannabinoids may be highly dependent on specific patient phenotypes and pain etiologies.

Ewing sarcoma is a rare, highly aggressive malignancy of bone and soft tissue, predominantly affecting children and young adults (Hesla et al. 2021). Pain is a very common and often severe symptom among patients with Ewing sarcoma, typically presenting as persistent, localized bone pain that may worsen at night or with physical activity. The pain is often described as deep and sharp, and it can persist for weeks or months, sometimes being mistaken for injury-related pain. Swelling or a palpable mass near the affected bone is also frequently reported (Heinemann et al. 2021).

Clinical studies specifically evaluating medical cannabis for Ewing sarcoma–related pain are, to our knowledge, lacking. However, a 2021 preclinical work demonstrates that Ewing sarcoma cell lines (TC-71, A-673) express non-canonical cannabinoid-binding sites; cannabinoids can induce cytotoxicity in EWS cell lines via non-canonical CBRs, which might be a potential therapeutic target to treat EWS (Shoeib et al. 2021). Exposure to phytocannabinoids or synthetic cannabinoids reduces cell viability and triggers cytotoxicity, suggesting testable biological plausibility for cannabinoid-mediated effects beyond analgesia.

Beyond putative antineoplastic actions, cannabinoids exhibit anti-inflammatory and putative antimicrobial properties relevant to complex pain states complicated by chronic infection. Medical cannabis can exert immunomodulatory effects via CB2 and non-CB pathways (e.g., TRPV1, adenosine A2A, PPAR-γ) and has shown in-vitro antibacterial activity against Staphylococcus aureus (including MRSA). While no cannabinoid is currently approved as an antibiotic and these data remain preclinical (Abrams 2022), such pleiotropic effects could hypothetically support symptom improvement in selected cases characterized by the convergence of nociceptive-inflammatory pain and recurrent soft-tissue infections.

Based on these hypotheses, we present the case of a young adult long-term survivor of Ewing sarcoma with refractory chronic pain and recurrent periprosthetic infections after multiple limb-salvage procedures, who experienced marked, sustained symptomatic improvement following initiation of medical cannabis. This report aims to detail the clinical course, contextualize observations within current guidelines, and acknowledge the limitations that preclude causal inference while motivating controlled studies in similarly complex scenarios.

## Case presentation

A 27-year-old male was diagnosed in September 2012 with Ewing sarcoma of the left proximal tibia, confirmed by histopathology and molecular identification of the *EWSR1–FLI1*fusion transcript. He was treated according to the ISG/AEIOP EW-1 protocol (Luksch et al. 2025), receiving multi-agent chemotherapy (vincristine, doxorubicin, ifosfamide, followed by busulfan and melphalan with autologous stem cell rescue) and wide surgical resection with reconstruction using a modular endoprosthesis.

In 2013, the implanted tibial endoprosthesis was removed for the first time due to mechanical complications. Postsurgical pain persisted and was severe (VAS 7–8) despite treatment with opioids (oxycodone and codeine).

The patient achieved complete oncologic remission, but the postoperative course was complicated by recurrent periprosthetic infections and multiple surgical revisions. From 2013 to 2024, he underwent more than ten orthopedic and plastic reconstructive procedures for septic loosening, fistulization, and soft-tissue necrosis. Chronic infection required repeated hospitalizations and long-term antibiotic regimens, including linezolid, dalbavancin, rifampicin, teicoplanin, minocycline, and levofloxacin. Over time, hepatotoxicity and antimicrobial resistance limited the available therapeutic options.

Persistent nociceptive and neuropathic pain developed at the surgical site, described as continuous and burning, radiating to the lower limb, with VAS scores of 9–10 despite the use of high-dose opioids (up to 120 mg/day morphine equivalents), paracetamol, and adjuvant analgesics. The patient reported sedation, nausea, and cognitive dulling from opioids, which significantly interfered with daily activities and social functioning.

**Fig. 1:**
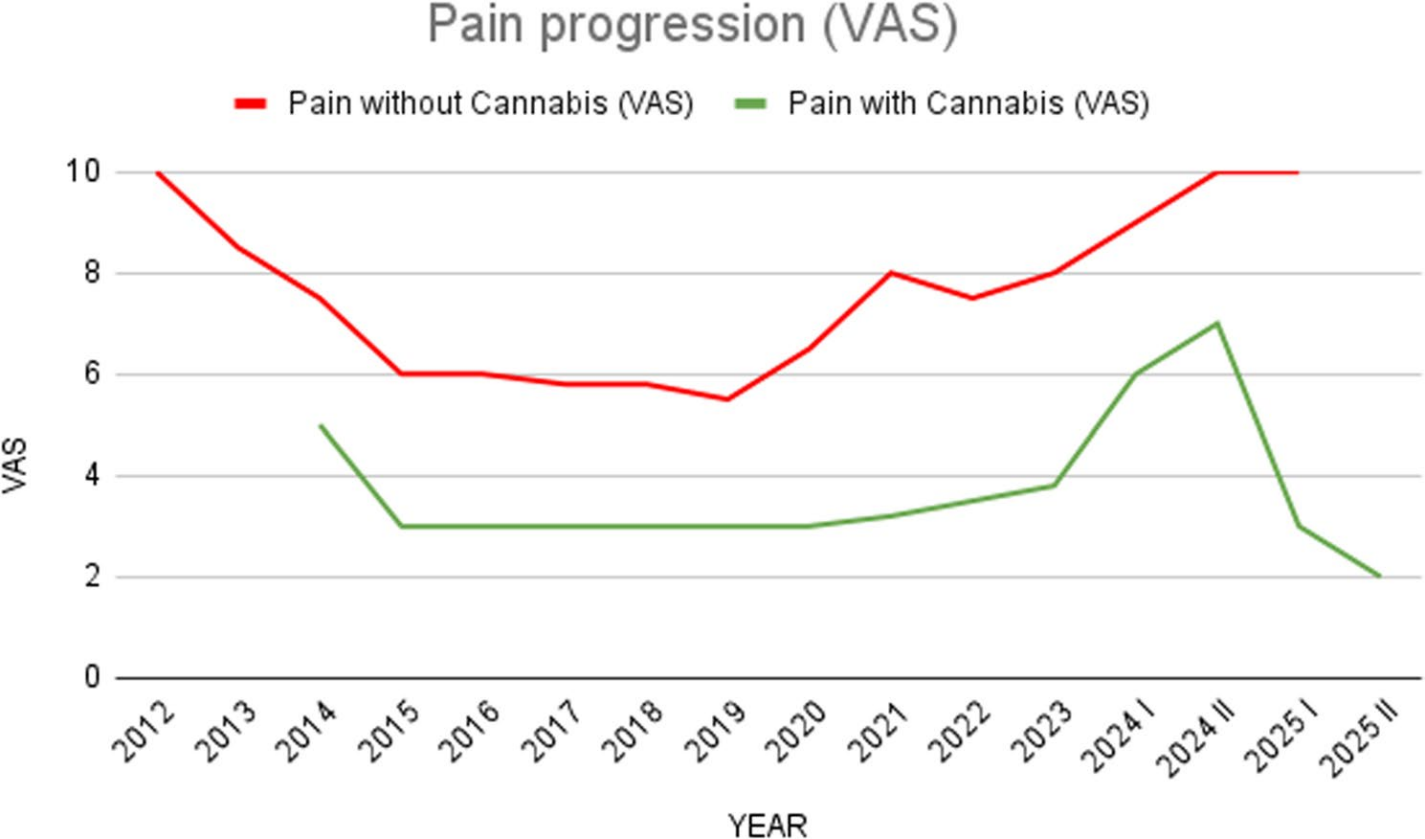
The graph shows the impact of cannabis in reducing patient pain compared to opioids

In 2014, due to persistent nociceptive and neuropathic pain that emerged after the second prosthetic surgery and seeking additional relief, the patient began self-administering cannabis in the form of inflorescences and decoctions (~ 2 g/day which he used as needed during the days of most intense pain). This assumption, occasional as it was limited by the patient’s ability to obtain the product, coincided with a reduction in pain intensity to VAS 4–5 and improved functional status, allowing partial ambulation with assistive devices (Fig. 1).

Between 2014 and 2020, he experienced a relatively stable phase with no additional surgeries, maintaining pain control mainly through cannabis use and minimal reliance on opioids.

From 2020 onward, progressive mechanical complications and recurrent infections required reoperations. During these phases, the patient used standard analgesic treatments, including high-dose morphine, oxycodone, and combinations of paracetamol and codeine, but pain control (even when combined) often remained insufficient, rarely reaching VAS scores below 6–7, when these agents were used alone (Fig. 1). Specifically during prosthetic replacements (November 2020) and complex surgeries (e.g., extensor revision in April 2021 and dorsal flap reconstruction in August 2022), optimal pain reduction to VAS 3 was achieved only when occasional self-provided cannabis was added to the standard opioid regimen (morphine and oxycodone), suggesting a synergistic effect or a specific response to cannabinoids where opioids failed (Fig. 1). Even in all subsequent débridements for infection (September 2021, February 2022, October 2023, November 2024), treated surgically and with antibiotics, only occasional cannabis use brought the patient’s pain below the moderate threshold, maintaining average pain levels on the VAS 3–4 and allowing acceptable management in the face of failure of other analgesics (Fig. 1).

**Fig. 2:**
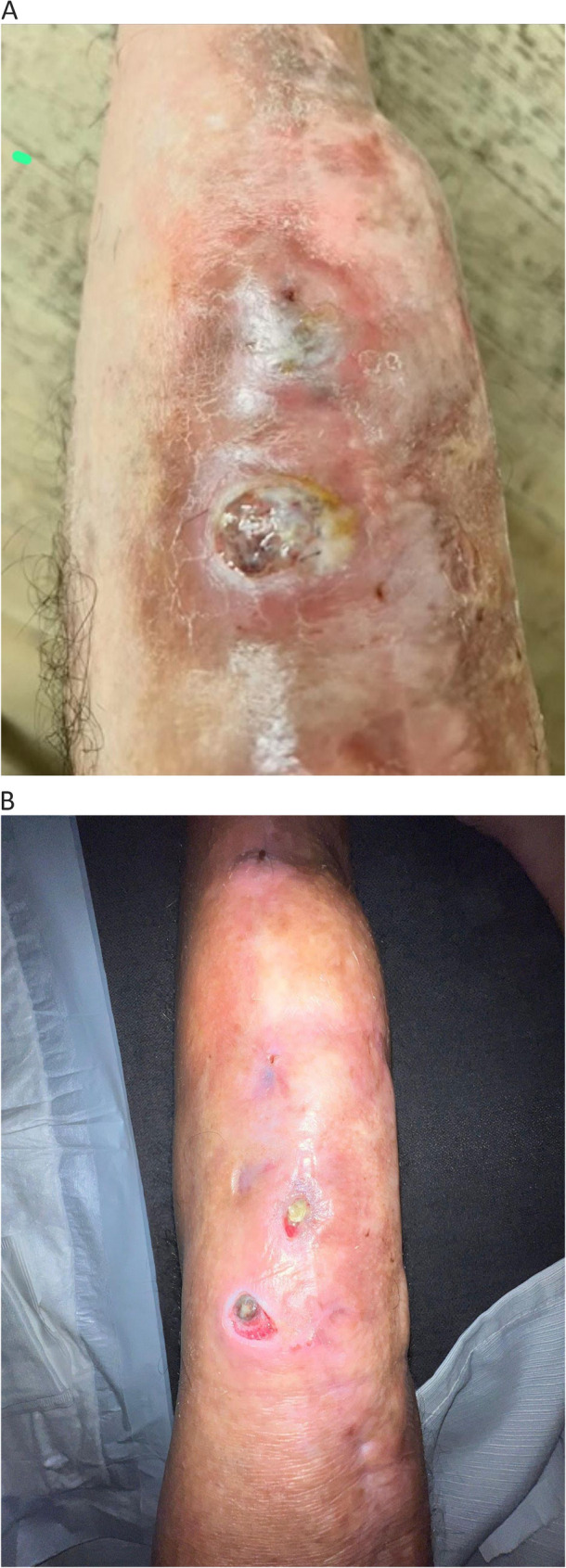
Clinical photographs of the left knee showing resolution of the chronic draining fistula and reduced periprosthetic inflammation. **A** Prior to medical cannabis initiation (January 2025); (**B**) After 8 months of medical cannabis therapy (September 2025)

In late 2024, due to recurrent fistulization and positive wound cultures for Staphylococcus aureus and Corynebacterium species, further debridement and antibiotic therapy (linezolid) were performed without sustained benefit. While the antibiotic treatment addressed the acute infectious flare, pain remained intolerable (VAS 9–10), despite taking high-dose morphine, oxycodone, and combinations of codeine and paracetamol. The clinical picture at this stage was characterized by the convergence of chronic inflammatory pain, neuropathic sensitization, and the sequelae of long-term opioid use (Fig. 2A). By January 2025, the patient was advised by the orthopedic team to consider limb amputation because of intractable pain and persistent infection. He refused the procedure and was referred to the Pain Therapy Clinic of ASL Bari for palliative management. At that time, he was taking oxycodone, paracetamol, and codeine, with limited analgesic efficacy and severe side effects. The pain prevented the patient from getting out of bed, resting at night, or carrying out basic daily activities with some functional autonomy.

In February 2025, patient was cared by Pain Clinic of the Bari Health Trust; supervised therapy with certified medical cannabis (Bedrocan^®^, 22% THC, 1% CBD, 1 g/day) was initiated. Pain rapidly decreased to a VAS of 2–3, coinciding with the complete discontinuation of opioid therapy. The closure of the cutaneous fistula and the normalization of inflammatory markers (CRP 9.6 to 2.3 mg/dL) were observed during the months following cannabis initiation. Although the patient had completed an antibiotic cycle shortly before, the sustained remission of infectious symptoms occurred under cannabis monotherapy. Pain remained stable at a VAS of 3.

By November 2025, the date of the last follow-up, the patient was in complete oncological remission with normalized inflammatory markers, full autonomy, and minimal pain (VAS of 2–3), while continuing cannabis monotherapy.

To ensure methodological consistency, pain intensity throughout the study was assessed using a 10-point Visual Analog Scale (VAS) for the ‘average pain over the last 24 hours’. Assessments were performed weekly during the titration phase and monthly during follow-up visits. To visualize the clinical progression and the temporal relationship between cannabis initiation and opioid tapering, a graphical representation of pain scores was developed (Fig. 1). 

### Clinical findings

At presentation, the patient was alert, cooperative, and fully oriented, with BMI 19.5 kg/m². He walked with crutches and wore a rigid knee brace to stabilize the left leg. The surgical scar over the proximal tibia appeared indurated with a small persistent draining fistula; there was no overt purulence. Local tenderness was marked, and the range of motion at the knee joint was limited (flexion < 30°). There was no peripheral edema or erythema extending proximally.

Systemic examination was unremarkable, and there were no signs of active tumor recurrence (Last CT scan performed at Rizzoli Hospital in Bologna in September 2025). The patient reported sleep fragmentation and fatigue secondary to nocturnal pain. Mood and cognitive screening (HADS, MoCA) showed mild anxiety but no depressive symptoms or cognitive impairment.

Pain assessment revealed a VAS score of 9/10 at rest and 10/10 during movement, with descriptors of burning, throbbing, and deep aching pain consistent with mixed nociceptive–neuropathic mechanisms. The Brief Pain Inventory (BPI) interference score averaged 8.7/10, highlighting a severe impact on activity, sleep, and mood.

## Diagnostic workup

When taking charge of Pain clinic, routine laboratory investigations showed elevated inflammatory markers (CRP 9.6 mg/dL, ESR 48 mm/h) but no leukocytosis. Liver function tests indicated mild transaminase elevation consistent with prior antibiotic-induced hepatotoxicity.

Microbiological cultures from the draining fistula repeatedly isolated *Staphylococcus aureus* (methicillin-susceptible and methicillin-resistant strains), *Enterococcus faecalis*, *Corynebacterium striatum*, and *Acinetobacter baumannii*. Antibiotic sensitivity testing confirmed multi-drug resistance to several β-lactams and macrolides, with retained susceptibility to linezolid and daptomycin.

Imaging studies included magnetic resonance imaging (MRI) of the left leg, which demonstrated cortical thickening, reactive bone edema, and signal abnormalities along the tibial endoprosthesis compatible with chronic periprosthetic osteomyelitis, but no evidence of tumor recurrence. A whole-body PET-CT scan showed no hypermetabolic lesions, confirming complete oncologic remission.

## Therapeutic intervention

Given the history of opioid resistance, hepatic intolerance to multiple antibiotics, and partial yet consistent benefit from long-term unsupervised cannabis use, the patient was enrolled in a supervised medical cannabis program at the Pain Therapy Clinic of ASL Bari in February 2025. The decision followed a multidisciplinary evaluation involving pain medicine, orthopedics, and infectious disease specialists.

A standardized cannabis flos preparation (Bedrocan^®^, The Netherlands) was prescribed. Each gram contained approximately 22% Δ⁹-tetrahydrocannabinol (THC) and 1% cannabidiol (CBD). The product was dispensed through a hospital pharmacy and administered either by vaporization (Bedrocan-compatible medical device) or decoction for oral intake. The patient began with a starting dose of 0.3 g/day, titrated over three weeks to a maintenance dose of 1 g/day (divided into two daily administrations, morning and evening), equivalent to approximately 50 mg of THC and 2 mg of CBD per day.

Concomitant medications—oxycodone, codeine, and paracetamol—were gradually tapered and discontinued within four weeks. No withdrawal symptoms were observed, potentially due to the overlapping antinociceptive effects of THC. The patient was educated on dosing, potential side effects, and safety precautions (e.g., avoiding driving within six hours after inhalation). Liver and renal functions were monitored monthly during titration.

Within the first two weeks, the patient reported a marked reduction in pain intensity from a baseline VAS 9–10 to VAS 3–4, with improved sleep quality and appetite. By the end of the first month, he was completely opioid-free, and pain stabilized at VAS 2–3, without breakthrough episodes requiring rescue analgesia (Fig. 1). He also reported significant improvement in energy levels, concentration, and mood, enabling the resumption of daily activities and university attendance.

Intermediate course (March–November 2025).

Over the following months, the patient continued daily cannabis therapy without adverse cognitive or psychiatric effects. He experienced no dizziness, sedation, or cardiovascular symptoms. Periodic evaluations documented sustained analgesia and improved physical function: he was able to ambulate independently with minimal use of assistive devices and engage in light physical activity.

Laboratory parameters showed progressive normalization of inflammatory markers (CRP decreased from 9.6 to 2.3 mg/dL; ESR from 48 to 18 mm/h) and stable hepatic and renal function. No new surgical interventions were required, and no antibiotic therapy was necessary during the 9-month follow-up. The chronic draining fistula showed gradual closure, and surrounding soft-tissue inflammation markedly subsided. Although a delayed effect of previous surgeries or antibiotics can’t be entirely excluded, the immediate and sustained stability observed only after starting cannabis monotherapy provides a compelling clinical correlation, especially given the chronic and refractory nature of the patient’s history.

## Follow-up and outcomes

The patient was followed for nine months after initiation of supervised medical cannabis therapy (February–September 2025). Follow-up visits were scheduled every four weeks for the first three months, then every two months. Each evaluation included clinical examination, pain assessment (VAS and BPI) (Fig. 1), monitoring of vital signs and neurocognitive status, and laboratory testing (complete blood count, liver and renal function, and inflammatory indices).

From the first month onward, the patient reported stable and sustained pain relief, with mean VAS scores of 2–3 and no need for additional analgesics. Intriguingly, opioid therapy was completely discontinued within four weeks of starting cannabis, and no withdrawal symptoms were observed. Functionally, the patient progressively regained autonomy in ambulation, transitioning from crutch-assisted gait to independent walking by the fourth month. Sleep quality and appetite improved markedly, and fatigue diminished.

Over the observation period, no new episodes of infection or surgical revision were required. The chronic fistulous tract of the left knee showed progressive healing with complete closure by month 8. (Fig. 2A e B). Serial laboratory results demonstrated a continuous decline in inflammatory markers (C-reactive protein from 9.6 to 2.3 mg/dL; ESR from 48 to 18 mm/h) and normalization of liver enzymes previously altered by long-term antibiotic exposure.

The patient did not experience adverse neuropsychiatric or cardiovascular effects. Mild transient dizziness during the first week of titration resolved spontaneously without dose adjustment. No cognitive impairment or psychomotor slowing was detected at routine follow-up.

At the last assessment (September 2025), the patient remained in complete oncologic remission and infection-free, reporting minimal pain (VAS 2–3) and a marked improvement in health-related quality of life (EQ-5D-5 L index 0.78, previously 0.42). Indeed, he resumed university studies, social activities, and independent living.

## Conclusions

This case report describes the notable response to medical cannabis treatment in a long-term patient with Ewing sarcoma, characterized by chronic, intractable, opioid-resistant pain and recurrent periprosthetic osteomyelitis. Administration of standardized cannabis flos (Bedrocan^®^, 22% THC, 1% CBD) was associated with sustained pain relief (reduction in VAS from 9–10 to 2–3) and allowed complete discontinuation of opioid therapy within four weeks, dramatically improving the patient’s function and quality of life. In addition to the analgesic effect, a significant regression of the clinical signs of chronic infection was observed, including complete healing of the draining fistula and normalization of systemic inflammatory markers (CRP and ESR) without further antibiotic or surgical therapy.

Despite the significant clinical improvement observed, several methodological limitations must be acknowledged to avoid overgeneralization. First, as a single case report, the observed association between cannabis initiation and clinical outcomes—particularly fistula closure and CRP normalization—cannot be definitively interpreted as causal. The natural history of chronic periprosthetic osteomyelitis, characterized by periods of remission, or the delayed effects of prior surgical debridements and long-term antibiotic regimens (e.g., linezolid, dalbavancin) could have contributed to the favorable outcome. Furthermore, potential confounding factors such as the “expectancy effect” cannot be ruled out, as the patient had previously experienced relief with self-administered cannabis. The improvement in functional status, sleep quality, and the psychological benefit of tapering sedative opioids likely created a synergistic effect that influenced the patient-reported pain scores (VAS). Lastly, while pre-clinical data support the antimicrobial and anti-inflammatory properties of cannabinoids (Abrams 2022; Gorzo et al. 2022), these findings remain laboratory-based and should not be extrapolated as evidence of clinical disease-modifying activity without further controlled trials.

Pain is frequently reported in survivors of Ewing sarcoma, for which the prognosis is improving. Different strategies have been experimented for the management of the pain, such as opioids, NSAIDs or nerve blocks (Wiffen et al. 2017; Fallon et al. 2018; Hesla et al. 2021; Heinemann et al. 2021). To our knowledge, no direct clinical trials specifically evaluate cannabis for pain in Ewing sarcoma patients, and our paper represents a rare documentation of this clinical experience. While recent meta-analyses have provided neutral or moderate-certainty evidence regarding the relief of opioid-refractory cancer pain by cannabinoids (Häuser et al. 2023), the rationale for the use in this case was related to the emerging role of cannabinoids in treating complex oncological pain phenotypes (Gorzo et al. 2022).

These findings, which contrast with the more cautious results shown in research on larger populations (Lichtman et al. 2018; Häuser et al. 2023; Fisher et al. 2021; Wang et al. 2021), suggest a potential pleiotropic therapeutic role for cannabinoids in the management of complex cancer pain syndromes, especially those aggravated by chronic inflammation. This emphasizes the need for controlled clinical trials aimed at defining the efficacy and mechanism of action of medical cannabis as an adjuvant therapy for refractory cancer pain, particularly in clinical settings complicated by chronic infections and high comorbidity.

## Data Availability

No datasets were generated or analysed during the current study.
